# Multi-environment genomic prediction for soluble solids content in peach (*Prunus persica*)

**DOI:** 10.3389/fpls.2022.960449

**Published:** 2022-10-06

**Authors:** Craig M. Hardner, Mulusew Fikere, Ksenija Gasic, Cassia da Silva Linge, Margaret Worthington, David Byrne, Zena Rawandoozi, Cameron Peace

**Affiliations:** ^1^Queensland Alliance for Agriculture and Food Innovation, The University of Queensland, Brisbane, QLD, Australia; ^2^Department of Plant and Environmental Sciences, Clemson University, Clemson, SC, United States; ^3^Faculty Horticulture, University of Arkansas System Division of Agriculture, Fayetteville, AR, United States; ^4^College of Agriculture and Life Sciences, Texas A&M University, College Station, TX, United States; ^5^Department of Horticulture, Washington State University, Pullman, WA, United States

**Keywords:** global genomic prediction, multivariate, G × E, mixed models, parsimony, factor-analytic

## Abstract

Genotype-by-environment interaction (G × E) is a common phenomenon influencing genetic improvement in plants, and a good understanding of this phenomenon is important for breeding and cultivar deployment strategies. However, there is little information on G × E in horticultural tree crops, mostly due to evaluation costs, leading to a focus on the development and deployment of locally adapted germplasm. Using sweetness (measured as soluble solids content, SSC) in peach/nectarine assessed at four trials from three US peach-breeding programs as a case study, we evaluated the hypotheses that (i) complex data from multiple breeding programs can be connected using GBLUP models to improve the knowledge of G × E for breeding and deployment and (ii) accounting for a known large-effect quantitative trait locus (QTL) improves the prediction accuracy. Following a structured strategy using univariate and multivariate models containing additive and dominance genomic effects on SSC, a model that included a previously detected QTL and background genomic effects was a significantly better fit than a genome-wide model with completely anonymous markers. Estimates of an individual’s narrow-sense and broad-sense heritability for SSC were high (0.57–0.73 and 0.66–0.80, respectively), with 19–32% of total genomic variance explained by the QTL. Genome-wide dominance effects and QTL effects were stable across environments. Significant G × E was detected for background genome effects, mostly due to the low correlation of these effects across seasons within a particular trial. The expected prediction accuracy, estimated from the linear model, was higher than the realised prediction accuracy estimated by cross-validation, suggesting that these two parameters measure different qualities of the prediction models. While prediction accuracy was improved in some cases by combining data across trials, particularly when phenotypic data for untested individuals were available from other trials, this improvement was not consistent. This study confirms that complex data can be combined into a single analysis using GBLUP methods to improve understanding of G × E and also incorporate known QTL effects. In addition, the study generated baseline information to account for population structure in genomic prediction models in horticultural crop improvement.

## Introduction

Genotype-by-environment interaction (G × E) is s common phenomenon in plant breeding (Allard and Bradshaw, 1964). Statistically, G × E may arise due to heterogeneity in variance and/or genetic correlation of less than one across environments (Baker, 1988; Burgueno et al., 2008). The consequence of significant G × E is that elite-performing germplasm in some environments is not necessarily elite in other environments.

Knowledge of G × E is important for designing breeding programs and deploying cultivars (Comstock and Moll, 1963; Cooper and Delacy, 1994; Harshman et al., 2016). If G × E is small, selection strategies will aim to identify germplasm with elite average performance (Hardner et al., 2019b; Kumar et al., 2019). This strategy may also be used where significant G × E is detected but no repeatable factor can be defined to classify and, hence, manage germplasm deployment. In contrast, germplasm may be targeted to specific environments if a repeatable factor explains some, or all, of G × E (Allard and Bradshaw, 1964; Cooper et al., 1996; Basford and Cooper, 1998).

Evaluation of G × E has commonly been undertaken using multi-environment trials (METs) of the connected germplasm (Malosetti et al., 2013). Advanced linear mixed model methods have been applied to combine data from multiple trials with different designs, repeated measures, and unbalanced replication within and across trials (Smith et al., 2005; Hardner et al., 2016, 2019a; Hardner, 2017). Accuracy of prediction in specific environments is commonly improved where data from multiple trials are combined (Hardner, 2017). Historically, the simple univariate genotype’s main effect plus genotype-by-environment interaction model is used to quantify G × E, although this approach is limited as uniform genetic variance and common pairwise correlation among all environments are essentially assumed (Smith et al., 2005). Multivariate models with specific genetic variance, and pairwise correlations, among pairs of environments, may improve the modelling of G × E (Smith et al., 2005; Malosetti et al., 2013). However, these models become complex as the number of environments increases, leading to over-parameterisation and difficulties in obtaining unique solutions for the G × E covariance matrices (Kelly et al., 2007). Solutions to these complex matrices can be obtained using factor-analytic parameterisation to model the major patterns in the covariance matrices with a reduced set of parameter matrices (Smith et al., 2001; Thompson et al., 2003; Kelly et al., 2007; Malosetti et al., 2013; Hardner, 2017).

While horticultural crops are planted around the world, knowledge of patterns of G × E in these crops is generally limited (Hardner et al., 2021). Where multi-environment trials have been used, clonal replication is usually employed to connect trials, but this can be expensive due to the large size of the experimental unit and the cost of assessing these units over several seasons (Peace et al., 2014; Hardner et al., 2021). Without information on the patterns of G × E, many horticultural tree breeding programs tend to have a local focus (e.g., Okie et al., 2008; Iezzoni et al., 2020) due to a lack of confidence in the performance in local target environments of germplasm developed in exotic environments. Local testing may also lead to little replication of the same germplasm among programs. Cost constraints may also mean many programs rely on unadjusted phenotypic observations of un-replicated germplasm to select new parents or advanced elite selections (Okie et al., 2008).

Genomic Best Linear Unbiased Prediction (GBLUP), which is a linear model that incorporates relationship matrices estimated from genome-wide genotypic data (genomic relationship matrices, GRMs), may offer a solution to exploring G × E patterns and improving confidence in the relative performance of exotic germplasm in local environments (Heslot et al., 2013; Hardner et al., 2021; Sneller et al., 2021). Essentially, genome-wide genotypic data enables the tracking of replicated chromosome segments across individuals. Therefore, the GRM models the overall genomic relatedness as well as the linkage disequilibrium between genetic markers and trait loci in a germplasm set (Habier et al., 2007). Commonly, additive genetic effects are modelled (Habier et al., 2007; VanRaden, 2008; Hayes et al., 2009b), but GRMs have been developed to model non-additive variation (Su et al., 2012). As GBLUP is an extension of standard linear mixed models, the flexibility of these mixed models can be exploited (Zhang et al., 2007; VanRaden, 2008; Heslot et al., 2012; Meuwissen et al., 2016).

Peach [*Prunus persica* (L.) Batsch] is the third-most important temperate fruit crop globally in terms of production and is consumed mainly as fresh fruit (Byrne et al., 2012; NASS USDA, 2017; FAO, 2020). Currently, peach production occurs in a wide range of adaptation zones, ranging from temperate high chill to subtropical and highland tropical low chill environments (Byrne, 2005; Okie et al., 2008; Luedeling, 2012). Although peach breeding programs develop cultivars for their specific adaptation zone, all new peach cultivars require market-specific fruit quality traits to be successful (Okie et al., 2008; Byrne et al., 2012; Cirilli et al., 2016). Peach breeding is undertaken using traditional phenotypic selection, but genotypic information is increasingly being incorporated (Peace, 2017). Genomic resources for peaches are available (Verde et al., 2012, 2013, 2017; Aranzana et al., 2019; Iezzoni et al., 2020), as well as the significant quantitative trait loci (QTLs) for many important quality traits that have been developed into DNA tests and used for selection (Eduardo et al., 2014; Sandefur et al., 2017; Vanderzande et al., 2018; Gasic and Saski, 2019; da Silva Linge et al., 2021; Fleming et al., 2022).

Sweetness is an important attribute supporting consumer demand for peach (Okie et al., 2008; Byrne et al., 2012; Delgado et al., 2013) and is a common selection priority in peach breeding (Byrne et al., 2012; Cirilli et al., 2016; Kelley et al., 2016). Consumer preference for peach fruit depends on the amount of total soluble sugars in ripe fruit (Crisosto et al., 2006; Cirilli et al., 2016). Broad and narrow sense heritability of sweetness measured as soluble solids content (SSC) in peach is reportedly very low (0.01) to moderate (0.47), as is the G × E (Byrne et al., 2012; Cirilli et al., 2016; Rawandoozi et al., 2020; da Silva Linge et al., 2021). Large effect QTLs for SSC and associated genetic markers in peach have been reported (Dirlewanger et al., 1999; Eduardo et al., 2011; Fresnedo-Ramirez et al., 2015; Hernandez Mora et al., 2017; Nunez-Lillo et al., 2019; Rawandoozi et al., 2020) with a large-effect QTL on chromosome 4 that is suggested to have a pleiotropic effect on SSC and ripening date (RD) (Eduardo et al., 2011).

This study evaluated the hypotheses that, by using GBLUP models, complex data from multiple breeding programs can be connected to improve knowledge of G × E for breeding and new cultivar deployment, and accounting for a known large-effect QTL improves the prediction accuracy. Hence, the objectives of this study were to (i) determine the magnitude of G × E for peach fruit sweetness measured as SSC and (ii) determine the improvement in prediction accuracy by accounting for the large-effect sweetness QTL on chromosome 4. The unbalanced dataset was from three US peach breeding programs with four trial locations and 2–3 seasons each with 577 accessions in total and 2–3 each with SNP array genotypic data for 4,473 SNPs.

## Materials and methods

### Plant material and phenotypic data

A total of 577 accessions (cultivars, breeding parents, progeny) from three breeding programs—Texas A&M University (TAMU) (Rawandoozi et al., 2021), University of Arkansas (UARK) (Worthington and Clark, 2021), and Clemson University (CLEM)—were evaluated for SSC (units: °Brix) for two or three seasons across four trial locations established in several US states (TAMU: Fresno CA, and College Station TX; UARK: Clarksville AR; CLEM: Seneca SC) (Iezzoni et al., 2020; Supplementary Table 1). The preliminary genotypic analysis identified four pairs of accessions with identical SNP-array DNA profiles (three pairs from UARK and one from CLEM), reducing the number of genetically unique individuals to 573 (Table 1).

At Fresno (F), 137 individuals were assessed for SSC in the 2011 and 2012 seasons, while 111 were assessed at College Station (G) in 2012 and 2013, with 104 individuals in common across these two TAMU trial locations (Table 1A). These individuals were in nine biparental F_1_ peach/nectarine families created from eight low to moderate chill nectarine/peach parents, with family sizes of 8–87 individuals. For the Fresno trial, SSC was assessed with a temperature-compensating refractometer of a composite sample for each individual in a season consisting of a macerated fruit pulp that was centrifuged to collect the juice from five fruit. At College Station, a hand-held refractometer was used to assess juice from individual fruit, and the average of 3–5 five fruits was recorded. Preliminary analysis identified that SSC data obtained from the composite and individual fruit protocols were highly correlated; thus, data were combined.

**TABLE 1A:** Number of peach/nectarine individuals within and among ten trial-by-season environments assessed for SSC from three breeding programs (Fresno and College Station: Texas A&M University (TAMU) population, Clarksville: University of Arkansas (UARK) population; and Seneca: Clemson University (CLEM) population).

Trial	Season within trial	Fresno (F)		College Station (G)		Clarksville (K)			Seneca (S)			SSC (°Brix)	
		2011	2012	2012	2013	2010	2011	2012	2010	2011	2012	Mean	Variance
Fresno (F)	2011 (F1)	103	100	36	79							11.9	5.5
	2012 (F2)	100	131	43	98							11.8	3.6
College Station (G)	2012 (G2)	36	42	49	45							12.3	3.3
	2013 (G3)	79	94	45	107							12.8	6.8
Clarksville (K)	2010 (K0)					54	54	52				12.1	4.4
	2011 (K1)					54	121	116				16.0	5.6
	2012 (K2)					52	116	125				17.1	10.1
Seneca (S)	2010 (S0)								87	82	26	11.5	4.2
	2011 (S1)								82	267	184	12.3	5.5
	2012 (S2)								26	184	215	11.7	2.9

At Clarksville (K), 133 accessions (130 unique individuals) were assessed for SSC in 2011, 2012, and 2013, while 302 individuals were assessed at Seneca (S) in the same seasons. No individuals at Clarksville or Seneca were evaluated in any other trial (Table 1A). Accessions at Clarksville consisted of parents and seedlings from six F_1_ peach families of 10–44 individuals, while accessions from Seneca comprised parents and seedlings of 12 F_1_ families of 6–22 individuals and three F_2_ families of 22–66 individuals. The SSC assessment method for Clarksville and Seneca followed the described (Frett et al., 2012; da Silva Linge et al., 2021) protocols. Ten fruits from each individual were harvested from the mid-canopy of each tree when they are slightly firmer than the ripe tree and placed into 0.24-L corrugated trays (FormTex Plastics Corp., Houston, TX, United States). A longitudinal slice was taken from each sample’s five largest fruits and juiced through a hand presser. Two to four drops of juice of the 5-fruit composite sample were measured for SSC using a refractometer (3810 PAL-1 Digital Hand-Held Pocket Refractometer, Atago Inc., Bellevue, WA, United States).

### Genotypic data

An initial set of 4,499 curated SNP data were obtained using the 9K peach SNP array (Verde et al., 2012) and published methods of curation (Vanderzande et al., 2019; da Silva Linge et al., 2021). The proportion of missing SNP genotypes per individual was low (0.2–4.5%) as was the proportion of missing genotypes per locus (0.4–2%). Twenty-six SNPs with a minor allele frequency lower than 0.05 were excluded. Genotypic data for a total of 4,473 SNPs were retained for downstream analysis (Supplementary Figure 1), representing an average marker density of 60 kbp per SNP (given a genome size of 265 mb, Yu et al., 2018). Missing alleles were imputed with BEAGLE software version 4.1 (Browning and Browning, 2007; Supplementary methods) to produce a complete sample-by-loci genotype table required by the downstream analyses. Unique QTL joint genotypes present among the accessions were defined for the eight SNPs within the 10,571,103–12,512,099 bp interval on chromosome 4 (Supplementary Figure 1), encompassing the region for the previously detected SSC QTL (Eduardo et al., 2011; da Silva Linge et al., 2021; Table 1B).

**TABLE 1B:** Number of defined unique joint genotypes, across a 2-Mb interval encompassing a large-effect QTL for SSC on chromosome 4 of peach, within and common across peach/nectarine germplasms evaluated at the four trials.

Trial	Fresno (F)	College Station (G)	Clarksville (K)	Seneca (S)
Fresno (F)	5	4	2	4
College Station (G)		4	2	4
Clarksville (K)			13	9
Seneca (S)				19

### Genome-wide genotypic structure of germplasm

Pairwise F_ST_ values among the populations from the three breeding programs were estimated to evaluate diversity among germplasm assessed across the three populations evaluated at the four trials. Pearson’s correlation coefficients were estimated to quantify the similarity of SNP allele frequencies for each breeding population with minor allele frequencies estimated across the three populations.

To evaluate the relationship between physical distance and linkage disequilibrium (LD) for the populations, squared correlation LD coefficients (R^2^) (Hill and Robertson, 1968) were estimated among SNP locus pairs using PLINK software version 9.1 (Purcell et al., 2007) and plotted against physical distance. A second-order, locally weighted scatterplot smoothing function (LOESS) (Esteras et al., 2013) was fitted to describe the decay in LD with physical distance.

### Linear models for G × E

Prior to model fitting, phenotypes on the original scale of assessment were scaled by trial-by-season phenotypic standard deviations to reduce the influence of heterogeneity in variance on the presence of G × E (Hill, 1984; Hardner, 2017). Total genomic effects were assumed to be composed of additive and dominance genomic effects with the estimated GRMs, according to VanRaden (2008) and Su et al. (2012), respectively. Where required, GRMs were made positive definite through bending (Jorjani et al., 2003; Nazarian and Gezan, 2016) as implemented in the R package ASRgenomics. An environment was defined with respect to the genomic effect (i.e., additive, dominance, or total) and was considered as a group of trial-by-seasons, among which genomic effects were homogenous (i.e., uniform genomic variance and genomic correlation of one). A full description of the GBLUP models used in this study is detailed in Supplementary methods.

A structured data-modelling strategy was undertaken to identify significant patterns and reduce the complexity of G × E for SSC in peach. In general, a univariate genomic main effect and genomic-by-environment interaction models were initially fitted to test the significance (p < 0.01) of genomic-by-environment interaction. A term for permanent environment effects was also included. Following this, multivariate genomic-by-environment models, where the expression of the phenotype in a specific environment was considered a different trait (following Falconer, 1952; Smith et al., 2001; Hardner et al., 2010), were used to identify significant G × E patterns. Cluster analysis (using Ward’s minimum distance) of the genomic-by-environment covariance matrix from these multivariate models was used to identify possible homogeneous environments (i.e., those that clustered together), and a reduced model (where these environments were constrained to be the same) was fitted and tested for a significant difference to the unconstrained model.

Restricted maximum likelihood (implemented in the R package ASReml v4, Butler et al., 2017) was used to estimate the random parameters of each model. Factor analytic parameterisation (Smith et al., 2001; Thompson et al., 2003) was used to estimate multi-dimensional genomic-by-environment covariance matrices. The significance of fixed effects was tested using the Wald tests (Kenward and Roger, 1997), and the significance of random effects was tested using log-likelihood testing, with appropriate adjustment for testing components at the boundary of the inference space (Stram and Lee, 1994). Akaike Information Criteria (Akaike, 1974) were also used to evaluate parsimony.

Single-trial genome-wide univariate (STGWU) models were fitted independently to data from each trial to test for significant within-trial G × E. GRMs for these analyses were estimated using only the genotypic data for the individuals at each specific trial. For trials where significant genomic-by-environment interaction was detected, single-trial genome-wide multivariate (STGWM) models were fitted to identify significantly unique genomic environments (i.e., combinations of seasons-within-trials, among which phenotypic variances due to genome effects were heterogenous and genomic correlations of these effects were less than 1) and, thereby, the most parsimonious single-trial models.

Multi-trial genome-wide univariate (MTGWU) models were fitted to the multi-trail data by combining the most parsimonious single-trial models to identify significantly unique genomic environments among trials. GRMs for these multi-trial models were estimated from the genome-wide genotypic data from all individuals across all trials. Pearson’s correlation was used to compare the off-diagonal elements of single population GRMs with the same elements in the multi-trial GRM. Where significant G × E was detected in the multi-trial univariate models, multi-trial genome-wide multivariate (MTGWM) models were fitted to identify significant across-trial G × E patterns and parsimonious multi-trial G × E models.

To evaluate the importance of QTL effects and study the interaction of QTL with the environment, genome-wide (additive and dominance) effects were separated into QTL and background effects. Separate GRMs were estimated for the QTL and background genomic effects using only the loci associated with each effect (i.e., the eight SNPs for the QTL GRM and the remaining 4,465 SNPs for the background GRM). A multi-trial QTL + background univariate (MTQBU) model was used to test for the significance of (additive and dominance) QTL-by-environment and background-by-environment interactions. Multi-trial QTL + background multivariate (MTQBM) models were then used to identify significant patterns in G × E and identify parsimonious models.

### Genomic architecture of soluble solids content

To evaluate the architecture of genomic effects for SSC, narrow- and broad-sense heritabilities were estimated for each trial-by-genomic environment (Supplementary methods). Phenotypic variation was estimated as the sum of genomic, permanent environment residual variances for the respective trial-by-environment. Genomic correlations among environments were estimated from the respective genomic variances and covariances. A cluster analysis using Ward’s (1963) minimum variance criteria of the Euclidean distance matrix transformation of the genomic effects correlation matrix was undertaken to visualise the genomic correlation among environments. A biplot (Kempton, 1984) of the environments (loadings) and individuals (scores) for the first two dimensions of the principal component reduction of the standardised genomic (additive or total, i.e., sum of additive and dominance effects) values-by-environment matrix was undertaken to visualise the major G × E patterns. Following principal component analysis (PCA), loadings were scaled by their respective variance, and scores were scaled to range from −1 to 1. The predicted performance of individuals in specific environments was obtained by projecting the performance of individuals onto the vectors for the environments for the respective genomic effects. Pearson’s correlation was used to compare the total-genome-effect predictions among models.

### Prediction accuracy

Expected prediction accuracy was estimated for additive and dominance genomic effects (genome-wide, QTL, and background) to evaluate the quality of genomic prediction models developed in this study. Three cohorts of individuals were constructed for each unique trial-by-genomic environment combination to evaluate the effect of the availability of phenotypic data on expected prediction accuracy. The first cohort for a specific trial-by-genomic environment included those individuals tested within trial-by-environment (TGE). The second cohort included those within the same trial but tested in a different genomic environment in that trial (Trial tested). The third cohort included individuals tested in another trial (Trial untested). Thus, expected prediction accuracy for the q^th^ genomic effect (additive/dominance by genome-wide/QTL/background) and the z_q_^th^ genomic environment at the l^th^ trial for the x^th^ cohort (TGE, Trial tested, Trial untested) was estimated as


$$rE_{q\left(l,z_{q},x\right)}=\sqrt{1-\frac{{\bar{\sigma }}_{i\left(q,l,z_{q},x\right)}^{2})}{v_{q\left(z_{q}\right)}}}$$

Realised prediction accuracies of additive and total genomic effects were estimated using five-fold cross-validation as the correlation between predicted genomic values in the validation population with their adjusted phenotypes (prediction ability), divided by the square-root of (narrow or broad-sense) heritability (Legarra et al., 2008; Muranty et al., 2015) estimated from the most parsimonious model. The realised prediction accuracy was also estimated for each trial-by-environment.

Two strategies were used to sample for validation populations. The first was a cross-trial sampling (XTCV), where individuals were sampled across all four trials to construct the validation population. This first strategy simulated performance prediction for new accessions based only on their genome-wide genotypic data. The second strategy was within-trial sampling (WTCV), where individuals were sampled within each trial, simulating performance prediction of accessions untested in a specific trial using their trained performance in other trials.

For each fold, phenotypic observations for the validation population were masked (i.e., set to missing) and the genomic values of the validation population were predicted by fitting the model of interest (including the full GRM containing all individuals in both the reference and validation populations). Adjusted phenotypes for the validation population were estimated by undertaking a full cross-trial model excluding any genomic terms (i.e., only trial, season, permanent environment effect, and residual) and summing the predicted permanent environment effect and the residual for each observational unit.

## Results

### Genome-wide genotypic structure of germplasm

Twenty-four unique QTL joint genotypes were identified. The majority (19) were identified in the germplasm assessed at Seneca, and only four were identified in the TAMU population evaluated at Fresno and College Station (Table 1B). Less genetic differentiation was detected between the CLEM population and the UARK population (F_*ST*_ = 0.075) than between the TAMU population and either the CLEM (F_*ST*_ = 0.104) or UARK (F_*ST*_ = 0.136) populations. There was a uniform distribution of minor allele frequencies between 0.15 and 0.45 across populations (Supplementary Figure 2). Allele frequencies, estimated using only genotypic data for the CLEM population, were highly correlated (0.85) with allele frequencies estimated across the entire study population (i.e., TAMU, UARK, and CLEM populations combined). The correlation of local allele frequencies across individuals in the UARK population with the entire population was lower (0.67) and lowest for the TAMU population (0.59) (Supplementary Figure 3A). The correlation between the off-diagonals of the population-specific additive GRM with off-diagonals of the GRM estimated using all individuals was higher for the CLEM population (0.98) than the UARK (0.96) or TAMU (0.93) populations (Supplementary Figure 3A).

**FIGURE 1:**
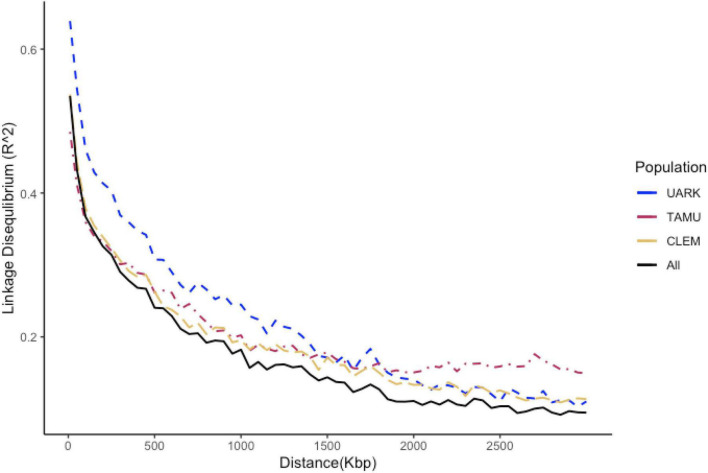
Decay trend of the linkage disequilibrium (LD) coefficient (R^2^) with a physical distance by the study population of peach/nectarine individuals assessed for SSC (UARK, University of Arkansas population; TAMU, Texas A&M University population; CLEM, Clemson university population; ALL, all populations combined). The vertical line is drawn at 30 kbp and the horizontal line at 0.42; the average LD across the entire set of peach/nectarine.

The average intra-chromosomal LD across the entire peach genome for the entire germplasm set was 0.38. Considerable variability among chromosomes was detected for LD (Supplementary Figure 4). LD decayed sharply with physical distance for the entire population with an average LD of 0.41 at 60 kpb (average marker density) (Figure 1). The distance among markers at 50% of maximum LD was 750 kpb across the entire population.

### G × E model fit

The main effect of season was significant for all trials (*p* < 0.001). No significant effect of the genome-wide dominance effects-by-season interaction on scaled SSC was detected for any of the STGWU models (Supplementary Table 2). The interaction between additive genome-wide effects and season was not significant at Fresno or College Station. The significant interaction between additive genome-wide effects and season at the Clarksville trial was associated with the contrast between the average of additive genomic effects across 2010 and 2011 (K01) against effects in 2012 (K2) (Supplementary Tables 2, 3A,B). The most parsimonious single-trial of a multivariate genome-wide model for accessions at Seneca contained heterogeneous additive genome-wide variances and unique pairwise additive genome-wide correlations for each assessment year (i.e., S0, S1, and S2) (Supplementary Tables 2, 3A,B).

A multi-trial univariate genome-wide model (MTGWU01) was successfully fitted to the full data sets of scaled SSC. Seven environments for additive genome-wide effects were defined for this model, following the results from the single-trial analyses (F, G, K01, K2, S0, S1, and S2). However, no significant dominance genomic-by-environment interaction was detected (MTGWU02, Supplementary Table 4). A multi-trial genome-wide model with a first-order factor analytic structure for the additive genome-wide environments (MTGWM02) was not significantly different from a higher-order model (MTGWM03) for the initial seven additive genome-wide environments. However, there was no significant difference between MTGWM02 and a model where the Clarksville 2010 and 2011 (K01) with Seneca 2012 (S2) were in a single environment (K01S2) (MTGWM07). No other reductions in complexity were identified.

Additive genome-wide by environment and dominance main genome-wide effects were successfully separated into effects associated with the QTL and with the background genome in a multi-trial univariate model (MTQBU01, Supplementary Table 5). However, no significant dominance QTL effects (MTQBU02 cf. MTQBU01), nor additive QTL-by-environment interaction (models MTQBU02 cf. MTQBU04), were detected. The fit of multi-trial multivariate models that attempted to reduce the complexity of the additive background genomic effect-by environment interaction was significantly poorer than the fit of MTQBMB1, which included six additive background genome environments (F, G, K01S2, K2, S0, and S1). As no significant G × E was detected for additive QTL and background dominance genome effects, the six additive genome-wide environments also defined the dimensions of the environments for total genome-wide effects.

### Genomic architecture of soluble solids content

Individual narrow-sense heritability for SSC estimated from the most parsimonious multi-trial QTL + background genome multivariate model varied between 0.57 and 0.72 among the seven trial-by-genomic environments (Fresno_F, College Station_G, Clarksville_K01S2, Clarksville_K2, Seneca_S0, Seneca_S1, and Seneca_K02S2) derived from the most parsimonious model (MTQBM91) (Table 2). Estimates of individual broad-sense heritability from the same model ranged from 0.66 to 0.80.

**TABLE 2:** Estimated model parameters (vAW, additive whole-genome variance; vAQ, additive QTL variance; vAB, additive background-genome variance; vDW, dominance whole-genome variance; vGW, total whole-genome variance; vU, within trial permanent among tree variance; vR, within trial tree-by-season residual variance; vP, phenotypic variance; h2, narrow sense heritability; H2, broad sense heritability) for most parsimonious single-trial genome-wide (STGW); multi-trial genome-wide (MTGW), or QTL + background (MTQB) multivariate models for SSC assessed on 577 peach/nectarine individuals across 10 trial-by-season environments.

Parameter	Model	Trial	Fresno	College Station	Clarksville		Seneca		
		GEnv	F	G	K01S2	K2	S0	S1	K01S2
vAW	STGW		0.728	0.000	0.762	0.891	0.530	0.428	0.236
	MTGW		0.908	1.247	0.552	0.696	0.434	0.469	0.552
	MTQB		1.082	1.245	0.740	0.746	0.768	0.714	0.740
vAB	MTQB		0.818	0.981	0.476	0.482	0.504	0.449	0.476
vAQ	MTQB		0.264	0.264	0.264	0.264	0.264	0.264	0.264
vDW	STGW		0.194	0.706	0.000	0.000	0.000	0.000	0.000
	MTGW		0.134	0.134	0.134	0.134	0.134	0.134	0.134
	MTQB		0.118	0.118	0.118	0.118	0.118	0.118	0.118
vGW	STGW		0.922	0.706	0.762	0.891	0.530	0.428	0.236
	MTGW		1.042	1.381	0.686	0.830	0.568	0.603	0.686
	MTQB		1.200	1.363	0.858	0.864	0.886	0.832	0.858
vU	STGW		0.144	0.175	0.000	0.000	0.046	0.046	0.046
	MTGW		0.054	0.000	0.000	0.000	0.039	0.039	0.039
	MTQB		0.010	0.211	0.000	0.000	0.053	0.053	0.053
vR	STGW		0.282	0.282	0.429	0.429	0.237	0.237	0.237
	MTGW		0.282	0.334	0.413	0.413	0.227	0.227	0.227
	MTQB		0.283	0.330	0.433	0.433	0.172	0.172	0.172
vP	STGW		1.348	1.163	1.191	1.320	0.813	0.711	0.519
	MTGW		1.378	1.715	1.099	1.243	0.834	0.869	0.952
	MTQB		1.493	1.904	1.291	1.297	1.111	1.057	1.083
h2	STGW		0.54	0.00	0.64	0.68	0.65	0.60	0.45
	MTGW		0.66	0.73	0.50	0.56	0.52	0.54	0.58
	MTQB		0.72	0.65	0.57	0.58	0.69	0.68	0.68
H2	STGW		0.68	0.61	0.64	0.68	0.65	0.60	0.45
	MTGW		0.76	0.81	0.62	0.67	0.68	0.69	0.72
	MTQB		0.80	0.72	0.66	0.67	0.80	0.79	0.79

Genomic environments (GEnv, where letters refer to trials and numbers refer to seasons) are defined as groupings of trial-by-seasons such that genomic variance is homogeneous, and genomic correlations are 1 within environments.

Additive genomic effects were the largest source of genomic variation (Table 2). The proportion of total genomic variance explained by additive effects varied between 86 and 91% for multi-trial QTL + background models and 76 and 90% for the multi-trial genome-wide models. The proportion of total genomic variance explained by additive genomic effects was more variable for the single-trial models as near-zero additive genomic variance was estimated for College Station and near-zero dominance variances were estimated for the Clarksville and Seneca trials. Additive QTL effects were estimated to account for between 21 and 37% of the additive genomic variance, 19–32% of total genomic variation, and 14–25% of phenotypic variation.

Correlations among total genomic effects predicted from single-trial and multi-trial, and genome-wide and QTL + background, multivariate models were higher (>0.87) than correlations among additive genomic effects (Table 3). Reflecting the relatively high correlation in total genomic effects predicted from the alternative multivariate models (0.87), there was little difference in the correlation of total genomic effects predicted from single-trial and multivariate models. On the other hand, correlations among additive genomic effects predicted from the single-trial or multi-trial models were more heterogeneous than for total genomic effects.

**TABLE 3:** Correlation among additive and total genomic effects predicted from single-trial G × E (Fresno, College Station, Clarksville, and Seneca) and multi-trial genome-wide (MTGW) and QTL + background (MTQB) multivariate models for SSC across four peach/nectarine breeding trials.

Model	Source	Additive		Total	
	Model	MTGW	MTQB	MTGW	MTQB
Fresno		0.94	0.94	0.96	0.96
College Station		0.75	0.77	0.93	0.94
Clarksville		0.97	0.93	0.98	0.97
Seneca		0.96	0.86	0.99	0.95
MTGW			0.79		0.87

**FIGURE 2:**
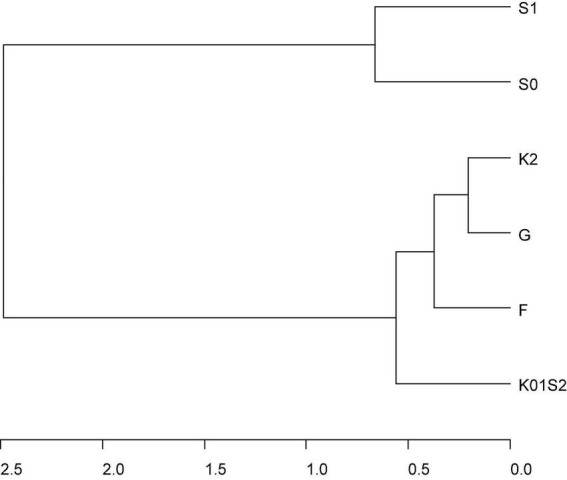
Cluster dendrogram of total genomic correlation matrix across genomic environments (F = Fresno 2011 and 2012, G = College Station 2012 and 2013, K01S2 = Clarksville 2010 and 2011 and Seneca 2012, S0 = Seneca 2010, S1 = Seneca 2011) estimated from the most parsimonious multivariate QTL + background genome model (MTQBM01, Supplementary Table 5) for SSC assessed across four peach/nectarine breeding trials. Genomic environments are defined as groupings of trial-by-seasons such that genomic variance is homogeneous, and genomic correlations are 1 within environments.

**FIGURE 3:**
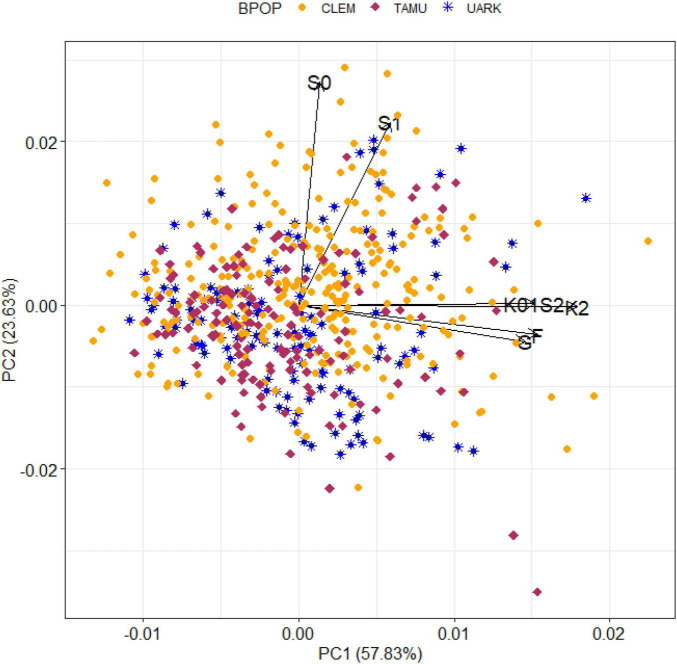
A biplot of the predicted total genomic effect for SSC of peach/nectarine individuals by genomic environment (defined in Figure 2) from the most parsimonious QTL + background genome model (MTQBM01).

There was a strong pattern in genomic correlations among environments across the various multi-trail G × E models for additive and total genomic effects (Figures 2, 3, Supplementary Table 6, and Supplementary Figures 5A–E); with a main group of environments defined by a large group of environments including all seasons at Fresno, College Station, and Clarksville, and the 2012 season at Seneca, and a looser grouping of genomic effects at Seneca in 2010 and 2011. Total genomic correlations for the most parsimonious QTL + background genomic effects model ranged from 0.66 to 1 within the main group, was 0.54 between 2010 and 2011 at Seneca and ranged between 0.06 and 0.33 among environments in across the two groups (Supplementary Table 6). These patterns were reflected in additive and total genomic correlations estimated with multi-trial genome-wide or QTL + background models (Supplementary Table 6). In general, genomic correlations were lower for additive genome-wide or background genome effects compared to total genomic effects.

The biplot (of standardised environmental loadings and scaled individual scores for the first two principal components of the decomposition of the total genomic-by-environment predicted effects for SSC from the most parsimonious QTL + background model) displayed the differential adaptation of individuals to genomic environments (Figure 3). Elite-performing individuals in each environment could be detected among all three breeding populations. For example, the two individuals with the highest predicted total genomic effect for SSC in the main group of environments (all seasons at Fresno, College Station, and Clarksville, and the 2012 season at Seneca) were from CLEM, while the third highest effect was from UARK and two individuals from TAMU had the eight and ninth highest values for this group of environments. Similarly, the 10 individuals with the highest predicted total genomic effect in the two Seneca environments originated from all three breeding programs. An accession from UARK was predicted to have high SSC in all environments except 2010 at Seneca.

### Prediction accuracy

The average expected prediction accuracy of additive genomic effects was higher than for dominance genomic effects (Table 4). In addition, the expected prediction accuracy of the cohort of genotypes tested at the respective trial-by-genomic environment (*TGE* cohort) was higher than for the cohort of individuals not tested at this trial-by-genomic environment (*Trial tested* and *Trial untested* cohorts) for all trial-by-genomic environments.

**TABLE 4:** Expected peach/nectarine SSC prediction accuracy (rE) of additive (A) and dominance (D) genomic effects for most parsimonious single-trial genome-wide (STGW), multi-trial genome-wide (MTGW), and multi-trial QTL + background genome (MTQB) multivariate models, and the number of individuals (n), by genomic environment (GEnv, for details, see Figure 2), within the trial for three test cohorts (TGE tested, individuals tested in the corresponding genomic environment within a trial; Trial tested, individuals tested in a different genomic environment at the corresponding trial; Trial untested, individuals not tested in the corresponding trial).

Parameter	Test cohort	Source	Model	Trial	Fresno	College Station	Clarksville		Seneca		
				GEnv	F	G	K01S2	K2	S0	S1	S2
rE	TGE	AW	STGW		0.87	–	0.91	0.90	0.71	0.76	0.78
		AW	MTGW		0.85	0.86	0.80	0.79	0.74	0.80	0.82
		AB	MTQB		0.84	0.84	0.78	0.75	0.78	0.82	0.83
		AQ	MTQB		0.97	0.97	0.96	0.96	0.97	0.86	0.97
		DW	STGW		0.49	0.81	–	–	–	–	0.70
		DW	MTGW		0.44	0.44	0.55	0.56	0.64	0.61	0.59
		DW	MTQB		0.45	0.45	0.55	0.56	0.65	0.62	0.61
	Trial	AW	STGW		na	na	0.82	0.81	0.51	0.60	0.55
		AW	MTGW		na	na	0.69	0.64	0.52	0.62	0.58
		AB	MTQB		na	na	0.66	0.59	0.56	0.63	0.59
		AQ	MTQB		na	na	0.97	0.94	0.96	0.97	0.97
		DW	STGW		na	na	–	–	–	–	–
		DW	MTGW		na	na	0.56	0.56	0.59	0.57	0.64
		DW	MTQB		na	na	0.57	0.56	0.60	0.57	0.64
	Untested	AW	STGW		na	na	na	na	na	na	na
		AW	MTGW		0.57	0.68	0.74	0.70	0.49	0.57	0.75
		AB	MTQB		0.56	0.67	0.75	0.68	0.54	0.59	0.73
		AQ	MTQB		0.97	0.97	0.97	0.97	0.97	0.97	0.97
		DW	STGW		na	na	na	na	na	na	na
		DW	MTGW		0.59	0.58	0.56	0.56	0.50	0.49	0.50
		DW	MTQB		0.60	0.59	0.57	0.57	0.50	0.50	0.50
*n*	TGE				134	111	121	125	87	267	215
	Trial				0	0	9	5	125	35	87
	Untested				439	462	443	443	271	271	271

Prediction accuracy from single-trial models for the cohort of individuals not tested at the respective trial could not be estimated. In addition, the accuracy of additive effects predicted with the single-trial model at College Station could not be estimated as the estimate of additive genomic variance in this model was near zero (Tables 2, 4). A similar situation occurred for dominance effects at Clarksville and Seneca.

There was little difference in the accuracy of predicted additive genome-wide or additive background genomic effects between multi-trial genome-wide and multi-trial QTL + background genome multivariate models (Table 4). There was also little difference in the expected accuracy of genome-wide dominance effects for either model. However, the expected prediction accuracy of QTL effects was consistently very high for all cohorts in all trial-by-genomic environments.

The difference between the expected accuracy of additive effects predicted from single-trial or multi-trial models was inconsistent among trial-by-genomic environments and cohorts (Table 4). For example, the prediction accuracy of additive genome-wide effects for the cohort of individuals tested within the respective trial-by-genomic environment (*TGE*, Table 4) was similar for single-trial and multi-trial models, except for the Clarksville trial where expected prediction accuracy was lower for the multi-trial models.

Greater differences were detected in the realised prediction accuracy among environments than among sampling methods or prediction models (Table 5). Prediction accuracy tended to be highest at Seneca, lowest at Clarksville, moderate for Fresno, and variable for College Station. The realised prediction accuracy was slightly higher for within-trial sampling compared to cross-trial sampling, particularly for the multi-trial models, but this was not consistent across models and trial-by-genomic environments. Prediction accuracy of additive effects ranged from 0.17 to 0.63 for single trial models and 0.25 to 0.70 for multi-trial models. The realised prediction accuracy of additive genomic effects was of similar magnitude to that for total genomic effects, except at Clarksville for single-trial models and the S1 and K01S2 genomic environments in the Seneca trial, particularly for single-trial models. Estimates of the realised prediction accuracy of additive genomic effects were higher for QTL + background models compared to single-trial models for most trial-by-genomic environments, except for the K02S1 environment at the Clarksville trial where prediction accuracy appeared lower. Differences in the realised prediction accuracy of additive effects between single-trial and multi-trial genome-wide models, and among all models for total genomic effects, were less consistent.

**TABLE 5:** Realised peach/nectarine SSC prediction accuracy for additive (A) and total (G) genomic effects, and the number of individuals in the validation population (*n*), for cross-trial (XT) or within-trial (WT) validation population sampling, predicted for single-trial genome-wide (STGW), multi-trial genome-wide (MTGW) or multi-trial QTL + background genome (MTQB) models by genomic environment (GEnv, for details, see Figure 2) within the trial.

Validation method	Model	Trial	Fresno			College Station			Clarksville						Seneca								
		GEnv	F			G			K01S2			K2			S0			S1			K01S2		
		Parameter	*n*	A	G	*n*	A	G	*n*	A	G	*n*	A	G	*n*	A	G	*n*	A	G	*n*	A	G
XT	STGW		27	0.49	0.48	22	0.17	0.34	24	0.49	0.49	25	0.40	0.40	17	0.61	0.62	53	0.44	0.60	43	0.48	0.60
	MTGW		27	0.48	0.47	22	0.26	0.25	24	0.40	0.40	25	0.40	0.39	17	0.61	0.62	53	0.51	0.59	43	0.56	0.61
	MTQB		27	0.51	0.50	22	0.36	0.34	24	0.41	0.43	25	0.44	0.45	17	0.67	0.68	53	0.57	0.64	43	0.67	0.69
WT	STGW		27	0.48	0.48	22	0.26	0.43	24	0.49	0.49	26	0.45	0.45	17	0.57	0.59	53	0.45	0.63	43	0.45	0.63
	MTGW		27	0.54	0.56	22	0.57	0.57	24	0.39	0.38	25	0.47	0.45	17	0.60	0.60	53	0.57	0.63	43	0.59	0.64
	MTQB		27	0.56	0.57	22	0.58	0.59	24	0.40	0.42	25	0.54	0.54	17	0.62	0.63	53	0.61	0.66	43	0.67	0.70

## Discussion

### Modelling strategy

This study extended and implemented multivariate GBLUP linear mixed models initially developed in cherry (Hardner et al., 2019b) to combine complex phenotypic data for SSC in peach assessed across multiple trials, with limited records of genetic connectedness among trials and unbalanced repeated assessment across seasons, and include dominance effects. Additive and dominance GRMs were successfully employed to connect multi-trial data, even though individuals were not replicated across trials, as the GRMs effectively tracked replicated genome segments across individuals. The linear mixed model framework employed here optimised the use of available data from unbalanced designs by weighting each observation by its correlation with each factor in the model (Henderson, 1963). Simpler models are expected to result in less optimal use of data (Hardner, 2017). Similar to our study, Biscarini et al. (2017) fitted within family repeated measures GBLUP models for peach fruit quality to accommodate non-genomic covariance of observations on the same tree, however, those models are not as general as the multi-family approach presented here.

This study has extended the structured approach for identifying significant parsimonious G × E models developed using pedigree relationship matrices (Hardner, 2017) to incorporate genomic relationship matrices. This approach contrasts with that used in other genomic prediction studies examining relative performance across multiple locations that assumed only a main genomic effect across locations (e.g., Hernandez Mora et al., 2017; da Silva Linge et al., 2021). Scaling observations by the phenotypic variance of each trial-by-season undertaken here to reduce the influence of variance heterogeneity in peach SSC on G × E (*sensu* Hill, 1984; Hardner, 2017) was not entirely successful as some unconstrained models were a significantly better fit to the data compared to those where highly correlated environments were constrained to be the same. A factor-analytic parameterisation (Smith et al., 2001; Thompson et al., 2003) was successfully implemented in this study to obtain estimates of model parameters for complex genomic-by-environment covariance matrices. The structured approach adopted in this study was preferable to an exhaustive naïve model of background genomic and QTL effects for both additive and dominance genomic effects for each of the 10 trial-by-season combinations, as the exhaustive model would be challenging to solve and interpret.

This study also implemented models to decompose anonymous genome-wide variation into the effect of a previously identified functional QTL and anonymous background genome variation. The higher than expected, and to some degree realised, prediction accuracy estimated here for the QTL + background model compared to a genome-wide anonymous model agrees with other studies (Bernardo, 2014; Bhandari et al., 2019). The inclusion of background effects was expected to have reduced confounding of the QTL effect with correlated unlinked SNPs (Lander and Schork, 1994; Yu et al., 2006; Bink et al., 2014). In addition, by treating the QTL region as random in this study—in contrast to treating it as a fixed effect (e.g., Bernardo, 2014)—over-estimation of QTL effects (e.g., Beavis, 1998) and bias due to dataset unbalance were expected to have been reduced. The successful implementation of a local genomic relationship matrix for the QTL region enabled information from related QTLs to be leveraged to improve accuracy and prediction of the effect of QTL genotypes in environments in which they were untested and therefore into other new environments in the future. Alternatively, QTL regions could have been modelled as haplotypes (Hernandez Mora et al., 2017) and a relationship matrix among the haplotypes incorporated. While the models used here assumed a Gaussian distribution of gene effects so that the flexibility of GBLUP models could be exploited, other models that incorporate a mixture of QTL allelic effect distributions were possible (Meuwissen et al., 2001; Erbe et al., 2012; Bink et al., 2014) but multivariate implementation has been challenging (e.g., Kemper et al., 2018).

### Discussion of study results

Estimates of narrow- and broad-sense heritability of sweetness (measured as SSC) reported here (0.6–0.7 and 0.7–0.8, respectively) were considerably higher than those reported in some previous studies (e.g., 0.02–0.33 in Brooks et al., 1993) but comparable to others (0.49—averaged over within family heritability estimates, Biscarini et al., 2017; 0.49, Hernandez Mora et al., 2017; 0.73—using essentially the same populations are these used in this study, da Silva Linge et al., 2021). Heritabilities reported in Rawandoozi et al. (2021) were not comparable here because of differences in approaches for estimating this parameter. The high heritability estimated in our study may be due to the large genetic diversity among parents. As expected (Su et al., 2012), estimates of dominance (and hence estimates of broad-sense heritability) in this study were less precise than estimates of additive genomic variance (and narrow-sense heritability). The higher heritability of the QTL + background model demonstrated the benefit of modelling the underlying genetic architecture of the trait (Meuwissen et al., 2001). Nevertheless, the genomic models employed here may not necessarily explain all variations (Yang et al., 2010). In addition, our results confirmed that the multi-trial estimates of heritability are less variable than the estimates from individual trial data.

While 50–70% of the total genomic variation of SSC in this study was due to the interaction of background genomic effects with the environment, the cluster and biplot results suggested genomic effects were stable across most environments sampled (Fresno, College Station, Clarksville, and 2012 season at Seneca—representing low- to high-chill environments) and that the major source of interaction was due to factors specific to the 2010 and 2011 seasons at the Seneca trial. In agreement with these results, Cantin et al. (2009) also reported that G × E for peach SSC was not significant. While moderate G × E in SSC between the Fresno and College Station environments using the same phenotypic data as this study was reported (Rawandoozi et al., 2021), the genetic models were not comparable. Other multi-trial studies (Biscarini et al., 2017; Hernandez Mora et al., 2017; da Silva Linge et al., 2021) did not directly model G × E. Further research is required to determine the cause of the differential performance of individuals at Seneca in 2010 and 2011. Our results also suggested that QTL and genome-wide dominance effects are expected to be stable across environments similar to those in this study, although the reduced precision in estimating dominance variance (discussed above) may also be a reason for this result.

This study confirmed the significant effect of a QTL on chromosome 4 for SSC in peach. Here, 20–30% of the total genomic variation was explained by the additive effect of the QTL, but significant dominance effects were not detected. Similarly, Eduardo et al. (2011) reported that this region explained 25% of the phenotypic variation in SSC in a single peach family, although only a minor (but significant) effect of this region was reported in other studies (Hernandez Mora et al., 2017; Rawandoozi et al., 2020) or was not detected (Fresnedo-Ramirez et al., 2015; Nunez-Lillo et al., 2019). No significant QTL × E interaction effects were detected, despite this QTL not being the most significant QTL in another study (Rawandoozi et al., 2020) using only the Fresno and College Station data. The absence of a significant QTL × E interaction here may be due to the limited segregation for this QTL in the TAMU population. Nevertheless, as the QTL was treated as random and the QTL segregated in correlated populations, the effect of all QTL genotypes was predicted for all environments, even for those in which they were untested.

The range of the realised prediction accuracy achieved in this study under the most parsimonious multi-trial QTL + background genome models (0.36–0.68) is considered (Hickey et al., 2014) relatively low. Biscarini et al. (2017) reported the realised prediction ability of 0.65–0.78 for peach SSC from fitting GBLUP models within individual full-sib families. This may be a consequence of trait architecture, prediction model, population size and structure, marker density, and validation strategy, as reviewed in other studies (reviewed Isidro et al., 2015; Crossa et al., 2017; Lebedev et al., 2020). However, higher marker density for these peach populations may not greatly increase prediction accuracy. This is because the LD of 0.41 at 60 kpb (average marker density) reported here is greater than an LD of 0.15 among adjacent markers suggested by Calus and Veerkamp (2007) as the minimum to capture all genetic variation in their study. Prediction accuracy in here may have been underestimated as the heritability estimates, used to adjust the correlation of the predicted genomic and phenotypic values, might be upwardly biased (as discussed above).

The difference between expected and realised prediction accuracies reported here indicated that these parameters assessed different characteristics of the genomic prediction models. The relatively high (0.75–0.97) expected prediction accuracy estimated in this study for additive, dominance, and QTL effects for individuals in the environments in which they were tested suggested that genetic effects are well-predicted, given the assumed genetic model is true. This assumption is similar to that made for the estimation of expected prediction accuracy in pedigree-based models which are based on laws of inheritance (Mrode, 2005). In contrast, the lower realised prediction accuracies reported in this study suggested that the assumption of a common GBLUP model of QTL and background effects across populations may not be the best model for prediction of untested individuals based only on genome-wide genotypic data. Similar to our results, Hayes et al. (2009a) reported expected prediction accuracy is lower than realised prediction accuracy for GBLUP models trained with multi-breed animal data. However, Hayes et al. (2009a) reported no difference between the two measures of prediction accuracy for models trained and applied within the same breed of animal in contrast to our results where differences were apparent even for single-trial models.

The higher expected, and in some cases realised, prediction accuracy, observed in this study demonstrated the value of using multi-trial models compared to single-trial models. Predictions from single-trial models only included those individuals tested at a particular trial; hence, the expected accuracy would be zero for untested individuals not included in the single-trial genomic relationship. In contrast, multi-trial models such as that developed here support the prediction of all individuals in all environments through genomic correlations among trials (Burgueno et al., 2012). The benefit of leveraging data from individuals in correlated trials was demonstrated by the higher expected prediction accuracy for individuals tested within a particular environment compared to predictions for individuals not tested in that environment. The higher realised prediction accuracy at Fresno and College Station (where individuals were replicated across trials) for multi-trial models compared to single-trial models for within-trial sampling for cross-validation (where individuals were replicated across trials) further demonstrates the benefit of correlated performance data. However, in agreement with previous studies (Burgueno et al., 2012; Krause et al., 2020), there was little enhancement in the realised prediction accuracy from multi-trial models where no phenotypic data were available (i.e., cross-trial sampling for the validation population).

This study highlighted the influence of population structure on estimates of the realised prediction accuracy. The major drivers of genomic prediction are short-range ancestral LD, co-segregation of linkage blocks within families, and general genome relationships, particularly among parents (Habier et al., 2013; Hickey et al., 2014). This study found evidence for divergent genetic structure among the breeding populations (*F*_*ST*_ values > 0.1, differences in LD among populations, and reduced correlation of within-population allele frequencies with overall allele frequencies). This structure was expected as the TAMU germplasm is focused on low-chill production while the focus of UARK and CLEM germplasm is on high-chill environments (Okie et al., 2008). In addition, the CLEM population was an admixture of alleles found in either the TAMU or UARK populations (da Silva Linge et al., 2021). However, estimates of th realised prediction accuracy in this study are unlikely to be upwardly biased by spurious correlations between unlinked markers and QTLs induced by historical selection history (*sensu* Yu et al., 2006; Toosi et al., 2010; Guo et al., 2014) as the breeding population was confounded with trial, and prediction accuracy was estimated for each trial-by-genomic environment. However, studies (Windhausen et al., 2012; Hickey et al., 2014; Werner et al., 2020) demonstrated that the realised prediction accuracy is driven by parental genetic values for training populations with strong family structures, such as in this and other horticultural tree crop studies (Kumar et al., 2012; O’Connor et al., 2021). While not undertaken here, training models within families (e.g., Biscarini et al., 2017) might avoid bias arising from strong family structure (Hickey et al., 2014; Werner et al., 2020), but such training might be expensive with little utility due to poor prediction accuracy in unrelated families (Riedelsheimer et al., 2013; Schopp et al., 2017). Training in a diverse unstructured population with high-density markers (e.g., Lorenz and Smith, 2015; Roth et al., 2020) is likely to better capture short-range ancestral LD between markers and functional QTLs.

The lack of consistent improvement in the realised prediction accuracy between single-trial and multi-trial models observed here might be explained by confounding testing location with population structure. The GRM for the single-trial models was estimated using only the local-germplasm genotypic data in contrast to the multi-trial GRM. However, allele frequencies differed between the single and combined populations. Thus, the single-trial models might have more accurately reflected the correlation between genomic effects of an individual and its phenotype compared to the multi-trial GRMs. In addition, the linkage phase between the marker and large-effect QTL alleles might be opposite across populations, hence reducing the correlation between marker and phenotype across a structured population. In contrast to our study, it is expected that the realised prediction accuracy would be improved if there was less differentiation among trials such as if some individual are replicated across trials (e.g., Hardner et al., 2019b). Prediction accuracy might also be improved if prediction models that explicitly account for population structure (Janss et al., 2012; Guo et al., 2014; Wientjes et al., 2017) are employed.

### Implications of study results

This study confirmed the hypothesis that the GBLUP models can be used to combine phenotypic data from multiple trials to support the genetic improvement of horticultural tree crops. In general, genomic models used in this study identified and dissected significant patterns in G × E for the deployment of elite germplasm, predicted additive and total genomic values for the selection of elite germplasm, and predicted genomic performance of untested germplasm in new environments. However, this study also identified population structure as a challenge for the use of genomic prediction models to combine data from different breeding programs.

Assuming the trials included in this study were representative of local production environments, the high genomic correlation of additive QTL background and dominance genomic effects among most environments observed here suggests attention to genotype-environmental matching for this trait is not required. However, other traits might influence the genomic expression of SSC in particular environments. For example, germplasm adapted to fruit production in high-chill environments similar to Clarksville, Fresno, and Seneca might not produce any fruit in low-chill environments, such as College Station, although introgression, particularly guided by genomic prediction, could support the development of locally adapted germplasm from exotic germplasm elite for particular traits (e.g., Kumar et al., 2020). A clearer understanding of the factors causing the poor genomic correlation of performance in 2010 and 2011 at Seneca with the other environments would improve confidence in deployment strategies.

The predictions of additive and total genomic values for individuals tested in this study could be used to select elite parents to produce new breeding populations (e.g., Kumar et al., 2020) and to select candidate cultivars for advanced testing and possible deployment in commercial orchards. The trials included in this study were similar to many others in horticultural tree crop improvement where only the phenotype of a single replicate of an individual is available to infer its genetic value. Given that heritability is the square of prediction accuracy, the expected prediction accuracy of individuals tested in a particular environment (TGE cohort in Table 4) is higher than the accuracy of the phenotypic section (estimated as the square root of heritability from the QTL + background model following Falconer, 1989). However, the advantage of genomic predictions over own phenotype is expected to be greater for traits with lower heritability compared to high-heritability traits such as SSC as information from genomic correlated individuals can be used to improve the performance of un-replicated individuals (Burgueno et al., 2012).

The relatively low estimates of the realised prediction accuracies for SSC in peach reported here suggested that the response to genomic selection for this trait may not be large (Hickey et al., 2014). However, these estimates may be low due to inflated individual heritability estimates. In addition, due to the family structure, our estimates of the realised prediction accuracy may more reflect the accuracy of predicting the breeding value of the parents used in this study rather than for genomic selection of individuals within families (*sensu* Hickey et al., 2014). On the contrary, our results, and those of others (Burgueno et al., 2012), suggest that genomic prediction would reduce experimental costs through unbalanced testing of individuals across environments (Krause et al., 2020) because prediction accuracy is higher for individuals tested in exotic environments but untested locally compared to individuals without any performance data.

Similar to the approaches undertaken here, Hardner et al. (2021) and others (Heslot et al., 2012; Hickey et al., 2014; Sneller et al., 2021) proposed using GBLUP models to connect data from breeding programs on a global scale to leverage additional value (i.e., improved prediction accuracy and increased selection intensity) from multiple available datasets. While our results confirm that there are benefits to improved understanding of patterns in G × E, prediction of untested germplasm into new environments, and increased prediction accuracy, the population structure needs to be better accounted to optimise this approach. This accounting could be through the use of less-structured germplasm that is more genetically related across testing locations and with denser genotyping than in the present study and/or statistical methods that account for population structure.

## Data availability statement

The data presented in this study are deposited in GDR data repository (https://www.rosaceae.org/publication_datasets) with accession number: tfGDR1062. The R-script for the analyses using these data has been deposited at GitHub (https://github.com/MulusewFikere/PeachGS), including the RData for the estimation of prediciton accuracy.

## Author contributions

CH performed the conceptualisation, data analysis, and preparation of the manuscript. MF contributed to data analysis and preparation of figures. KG performed the data collection, interpretation of results, manuscript review, and funding acquisition. CS wrote and reviewed the manuscript. MW performed the interpretation of results, wrote and reviewed the manuscript. DB performed the interpretation of results, manuscript review, and funding acquisition. ZR performed the data collection and wrote and reviewed the manuscript. CP performed the conceptualisation, interpretation of results, and wrote and reviewed the manuscript. All authors contributed to the article and approved the submitted version.
