# Supplementary material for: Multi-environment genomic prediction for soluble solids content in peach (Prunus persica)
Source: Front Plant Sci. 2022 Oct 6;13:960449. doi: 10.3389/fpls.2022.960449 (PMC9583944; doi:10.3389/fpls.2022.960449)
Supplement: Supplementary file 1 [file Data_Sheet_1.docx]

Multi-environment genomic prediction for soluble solids content in peach (*Prunus persica*)

Craig M. Hardner^1^, Mulusew Fikere^1,2^, Ksenija Gasic^3^, Cassia da Silva Linge^3,4^, Margaret Worthington^5^, David Byrne^6^, Zena Rawandoozi^6^, Cameron Peace^7^

# SUPPLEMENTARY MATERIAL

## Supplementary methods

### Details of Beagle

The command line for the imputation of missing markers was

beagle java -Xmx30g -jar /usr/local/beagle/4.0/beagle-4.0.jar gl=InputFile.vcf nthreads=1 out=outputFileName

No reference panel or pedigree was used. As our genotypic data set is quite small, default values for parameters were used (number of iteration = 12, size of sliding window = 40.0)

### Details of Linear models

#### Genomic model

The genomic effects model for the effect of the *i*^th^ individual in the *z_g_*^th^ environment for total genomic effects was defined as:

$g_{(i,z_{g})}=a_{(i,z_{g})}+d_{(i,z_{g})}$’

where

$a_{(i,z_{g})}$ was the additive genomic effect of the ith individual in the *z_g_*^th^ environment and

$d_{(i,z_{g})}$ was the dominance genomic effect of the ith individual in the *z_g_*^th^ environment

#### General formulation of linear mixed model

##### Univariate models

Assuming a common genomic variance across genomic environments, and homogeneous genomic effect-by-environment covariance among environments, the general model for the univariate models was

where

$y_{\left(ljk\right)}$was the scaled observation at the *l*^th^ trial for the *j*^th^ tree in the *k*^th^ season,

$ts_{\left(lj\right)}$was the fixed combined effect of the *j*^th^ season at the *l*^th^ trial,

$a_{\left(i\right)}$ was the random main additive genomic effect of the *i*^th^ individual across environments (with variance $MVN(\mathbf{0},\mathbf{A}\otimes\sigma_{a}^{2})$ where $\mathbf{A}$ was the additive genomic relationship matrix and $\sigma_{a}^{2}$ was the additive genomic variance),

$ae_{\left(i,z_{a}\right)}$ was the random specific additive genomic effect of the *i^th^* individual in the *z_a_*^th^ additive genomic environment not explained by the main additive genomic effect (with variance $MVN(\mathbf{0},\mathbf{A}\otimes\sigma_{ae}^{2})$ where $\mathbf{A}$ was as defined above, and $\sigma_{ae}^{2}$ was the variance of specific additive genomic-by-environment effects),

$d_{\left(i\right)}$ was the random main dominance genomic effect for the *i*^th^ individual across environments (with variance $MVN(\mathbf{0},\mathbf{D}\otimes\sigma_{d}^{2})$ where $\mathbf{D}$ was the dominance genomic relationship matrix and $\sigma_{d}^{2}$ was the dominance genomic variance),

$de_{\left(i,z_{d}\right)}$ was the random specific dominance genomic effect of the *i*^th^ individual in the *z_d_*^th^ environment for dominance genomic effects not explained by dominance genomic main effects (with variance $MVN(\mathbf{0},\mathbf{D}\otimes\sigma_{de}^{2})$ where $\mathbf{D}$ was the dominance genomic relationship matrix, and $\sigma_{de}^{2}$ was the variance of specific dominance genomic-by-environment effects),

$u_{\left(lj\right)}$ was the non-genomic effect of the *j*^th^ tree at the *l*^th^ trial (with variance at the *l*^th^ trial $MVN(\mathbf{0},\mathbf{I}_{u(l)}\otimes\sigma_{u(l)}^{2})$ where $\mathbf{I}_{u(l)}$ was an identity matrix with dimension equal to the number of trees at the *l*^th^ trial and $\sigma_{u(l)}^{2}$ is the variance among the non-genomic tree effects), and

$r_{\left(ljk\right)}$ was the unexplained residual effects for the *j*^th^ tree at the *l*^th^ trial in the *k*^th^ season (with variance $MVN(\mathbf{0},\mathbf{I}_{r(l)}\otimes\sigma_{r(l)}^{2})$ where $\mathbf{I}_{r(l)}$ was an identity matrix with dimension equal to the total number of observations across seasons at the *l*^th^ trial and $\sigma_{r(l)}^{2}$ was the residual variance at the *l*^th^ trial).

##### Multivariate model

The general model for the multivariate models was

where

$y_{\left(ljk\right)}$was the scaled observation at the *i*^th^ trial for the *j*^th^ tree in the *k*^th^ season,

$ts_{\left(ik\right)}$was the fixed combined effect of the *k*^th^ season at the *i*^th^ trial,

was the random additive genomic effect of the *i*^th^ individual in the *z_a_*^th^ environment for additive genomic effects (with variance $MVN(\mathbf{0},\mathbf{A}\otimes\mathbf{E}_{a})$, where $\mathbf{A}$ was as defined above, $\mathbf{E}_{a}$ was the additive-by-environment genomic co-variance matrix, and indicates a Kronecker product),

was the random dominance genome effect of the *i*^th^ genotype in the *z_d_*^th^ environment for dominance genomic effects (with variance $MVN(\mathbf{0},\mathbf{D}\otimes\mathbf{E}_{d})$ where $\mathbf{D}$ was defined as above and $\mathbf{E}_{d}$ was the dominance genomic-by-environment covariance matrix), and

$u_{\left(lj\right)}$ and $r_{\left(ljk\right)}$ were as defined above for the univariate model.

##### Estimation of genomic relationship matrices

Scaled and centred additive genomic relationship matrices were estimated as (Van Raden, 2008; Endelman and Jannink, 2012; Poland et al., 2012):

$$\mathbf{A}=\frac{\mathbf{ZZ}^{T}}{\sum2p_{m}(1-p_{m})}$$

where

*p_m_* was the minor allele frequency of the *m^th^* locus across the population, and

**Z** was the centred genotype-by-marker incidence matrix estimated as

$$z_{(i.m)}=x_{(i,m)}-2p_{m}$$

where

$x_{\left(i,m\right)}$ is the count of reference alleles at the *m^th^* locus for the *i^th^* individual.

Dominance genomic relationship matrices were estimated as (Su et al., 2012):

$$D=\frac{\mathbf{HH}^{T}}{\sum2(1-2p_{m}^{2}(1-p_{m})^{2})}$$

where

$$h_{(i,m)}=2p_{m}(1-p_{m})\text{ if }x_{(i,m)}=0\text{ or 2}$$

or

$h_{(i,m)}=1-2p_{m}(1-p_{m})\text{ if }x_{(i,m)}=1$.

Random additive and dominance genome-wide relationship matrices (**AW**, **DW**) were estimated using all available markers across the genome. For single-trial models, genomic relationship matrices were estimated using only genotypic data for the individuals at the respective trial.

Genotypes of the eight markers in the known SSC QTL region was used to model variation for the QTL as local random additive and dominance effects with additive and dominance genomic relationship matrices (**AQ**, **DQ**). Relationship matrices for additive and dominance background genomic effects were estimated using the remaining markers (**AB**, **DB**).

#### Estimation of heritability

Individual narrow- and broad-sense genomic heritabilities were estimated for the *l^th^* environment at the *i^th^* trial as

$$\hat{h}_{(il)}^{2}=\frac{\hat{v}AW_{(l)}}{\hat{v}AW_{(l)}+\hat{v}DW_{(l)}+\hat{v}U_{(i)}+\hat{v}R_{(i)}}$$

$$\hat{H}_{(il)}^{2}=\frac{\hat{v}AW_{(l)}+\hat{v}DW_{(l)}}{\hat{v}AW_{(l)}+\hat{v}DW_{(l)}+\hat{v}U_{(i)}+\hat{v}R_{(i)}}$$

where

$\hat{v}AW_{(l)}$ was the estimated additive genome-wide variance for the *l^th^* environment

$\hat{v}DW_{(l)}$ was the estimated dominance genome-wide variance for the *l^th^* environment

$vU_{(i)}$ was the estimated permanent environment variance for the *i^th^* trial, and

$vR_{(i)}$ was the estimated residual variance for the *i^th^* trial

For QTL + Background genome models $vAW_{(l)}$ was estimated as the sum of additive QTL variance at the *l^th^* environment ( $vAQ_{(l)}$) and additive background genomic variation at the *l^th^* environment ($vAW_{(l)}$).

## Tables

**Supplementary Table 1 |** Agroecological characteristics of four trial sites of peach/nectarine individuals assessed for SSC

|  |  |  |  |  |
| --- | --- | --- | --- | --- |
| Trial | Lat | Climate | Soil Type | Management |
|  |  |  |  |  |
|  |  |  |  |  |
| Fresno, CA (F) | 36 44'52''N,  119 46'21''W | Semi-arid Mediterranean | Alluvial sandy-loam | Y scaffold, 1 m × 4 m |
|  |  |  |  |  |
| College Station, TX (G) | 30 36'5''N,  96 18'52''W | Sub-humid, warm temperate, mild winters and warm to hot summers | Clay-pan | Central leader, 1.7 m × 0.7 m, double rows 5 m apart |
|  |  |  |  |  |
| Clarksville, AR (K) | 35°31’58’’N 93°24’12’’W | Sub-humid, warm temperate, cold winters and warm to hot summers | Linker fine sandy loam (Typic Hapludult) | open-center trained and spaced 5.5 m between trees and rows or trained to a perpendicular-V system with trees spaced 1.9 m in rows spaced 5.5 m apart |
|  |  |  |  |  |
| Seneca, SC (S) | 34º 38’20” N  82º 56’6” W | Sub-humid, warm temperate, cold winters and warm to hot summers | Sandy loam | Modified Spanish open centre, 2.5 × 6.7 m |
|  |  |  |  |  |

**Supplementary Table** **2 |**Log-likelihood (log), degrees of freedom (df) and Akaike Information Criterion (AIC) for the fit to peach/nectarine SSC of single-trial (F = Fresno, G = College Station, K = Clarksville, S = Seneca) nested univariate (U) or multivariate (M) genotype-by-environment interaction models (G×E mod) of additive (Amod) and dominance (Dmod) genome-wide effects. Additive and dominance genomic environments (AWE and DWE respectively) defined by groupings of seasons (where the letter defines the trial and the numbers define the season of assessment) so that genetic variance was assumed homogenous, and genetic correlations were 1, within environments. Genomic relationship matrices for each trial were estimated using genotypic data from only those entries at that trial. Fxed season effects, and random individual tree and tree-by-season residual effects were also fitted for each trial. The notation *fa1* (factor analytic order 1) and *us* (unstructured) refer to the structure of fitted genotype-by-environment variance-covariance matrix.

|  |  |  |  |  |  |  |  |  |  |  |  |  |
| --- | --- | --- | --- | --- | --- | --- | --- | --- | --- | --- | --- | --- |
| ID | Trial | G×E mod |  | Amod | AWE | Dmod | DWE | Rmod |  | logl | df | AIC |
|  |  |  |  |  |  |  |  |  |  |  |  |  |
|  |  |  |  |  |  |  |  |  |  |  |  |  |
| FU01 | F | U |  | AWF + AWF:AWFE1 | F1, F2 | DWF + DWF:DWFE1 | F1, F2 | UF + RF |  | -69.0 | 3 | 144 |
| FU03 | F | U |  | AWF |  | DWF |  | UF + RF |  | -69.0 | 3 | 144 |
| FU04 | F | U |  | AWF |  |  |  | UF + RF |  | -71.0 | 3 | 148 |
|  |  |  |  |  |  |  |  |  |  |  |  |  |
| GU01 | G | U |  | AWG + AWG:AWGE1 | G2, G3 | DWG + DWG:DWGE1 | G2, G3 | UG + RG |  | -65.5 | 3 | 137 |
| GU02 | G | U |  | AWG + AWG:AWGE1 | G2, G3 | DWG |  | UG + RG |  | -65.5 | 3 | 137 |
| GU03 | G | U |  | AWG |  | DWG |  | UG + RG |  | -65.6 | 2 | 135 |
| GU04 | G | MV |  | AWG |  |  |  | UG + RG |  | -68.0 | 3 | 142 |
|  |  |  |  |  |  |  |  |  |  |  |  |  |
| KU01 | K | U |  | AWK + AWK:AWKE1 | K0, K1, K2 | DWK + DWK:DWKE1 | K0, K1, K2 | UK + RK |  | -103.1 | 5 | 216 |
| KU02 | K | U |  | AWK + AWK:AWKE1 |  | DWK |  | UK + RK |  | -104.2 | 4 | 216 |
| KU03 | K | U |  | AWK |  | DWK |  | UK + RK |  | -112.0 | 2 | 228 |
| KU05 | K | U |  | AWK + AWK:AWKE1 |  |  |  | UK + RK |  | -104.2 | 4 | 216 |
| KM02 | K | MV |  | AWK:*fa1*(AWKE1) | K0, K1, K2 | DWK |  | UK + RK |  | -103.5 | 8 | 223 |
| KM02a | K | MV |  | AWK:*us*(AWKE1) | K0, K1, K2 | DWK |  | UK + RK |  | -103.5 | 8 | 223 |
| KM06 | K | MV |  | AWK:*us*(KWE2) | K01, K2 | DWK |  | UK + RK |  | -104.5 | 5 | 219 |
|  |  |  |  |  |  |  |  |  |  |  |  |  |
| SU01 | S | U |  | AWS + AWS:AWSE1 | S0, S1, S2 | DWS + DWS:DWSE1 | S0, S1, S2 | US + RS |  | -137.0 | 6 | 286 |
| SU02 | S | U |  | AWS + AWS:AWSE1 | S0, S1, S2 | DWS |  | US + RS |  | -137.2 | 5 | 284 |
| SU03 | S | U |  | AWS |  | DWS |  | US + RS |  | -193.5 | 3 | 393 |
| SU05 | S | U |  | AWS + AWS:AWSE1 | S0, S1, S2 |  |  | US + RS |  | -138.1 | 4 | 284 |
| SM02 | S | MV |  | AWS:*fa1*(AWSE1) | S0, S1, S2 | DWS |  | US + RS |  | -136.7 | 9 | 291 |
| SM02a | S | MV |  | AWS:*us*(AWSE1) | S0, S1, S2 | DWS |  | US + RS |  | -136.7 | 9 | 291 |
| SM06 | S | MV |  | AWS:*us*(AWSE2) | S0, S1 | DWS |  | US + RS |  | -175.5 | 5 | 361 |
|  |  |  |  |  |  |  |  |  |  |  |  |  |

**Supplementary Table** **3a |** Estimates of variance components (vA = additive genomic, vAY = additive genomic-by-year, vD = dominance genomic, vU = permanent tree non-genomic, vR = residual) of most parsimonious univariate genome-wide model for SSC of peach/nectarine individuals using only single trial (F = Fresno, G = College Station, K = Clarksville, S = Seneca) phenotypic data and genomic relationship matrices constructed using genotypic data for only germplasm present in trial.

|  |  |  |  |  |  |  |
| --- | --- | --- | --- | --- | --- | --- |
| Trial | Model | vA | vAY | vD | vU | vR |
|  |  |  |  |  |  |  |
|  |  |  |  |  |  |  |
| F | FU03 | 0.000 |  | 0.729 | 0.069 | 0.282 |
| G | GU03 | 0.000 |  | 0.907 | 0.000 | 0.356 |
| K | KU02 | 0.197 | 0.351 | 0.000 | 0.096 | 0.440 |
| S | SU02 | 0.294 | 0.267 | 0.078 | 0.047 | 0.303 |
|  |  |  |  |  |  |  |

**Supplementary Table** **3b |** Estimated parameters (vA = additive genomic variance, rA = additive genomic correlation, vD = dominance genomic variance) for most parsimonious single-trial multivariate multi-season (Y) model for SSC of peach/nectarine individuals using only single trial (K = Clarksville, S = Seneca) phenotypic data and genomic relationship matrices constructed using genotypic data for only germplasm present in trial.

|  |  |  |  |  |  |  |  |
| --- | --- | --- | --- | --- | --- | --- | --- |
| Trial |  |  | vA | rA |  |  | vD |
|  | Y |  |  | 2011 | 2012 |  |  |
|  |  |  |  |  |  |  |  |
|  |  |  |  |  |  |  |  |
| K | 2010 |  | 0.381 | 1 | 0.48 |  | 0.000 |
|  | 2011 |  | 0.381 |  | 0.48 |  | 0.000 |
|  | 2012 |  | 0.603 |  |  |  | 0.000 |
|  |  |  |  |  |  |  |  |
|  |  |  |  |  |  |  |  |
| S | 2010 |  | 0.591 | 0.66 | 0.50 |  | 0.082 |
|  | 2011 |  | 0.554 |  | 0.47 |  | 0.082 |
|  | 2012 |  | 0.609 |  |  |  | 0.082 |
|  |  |  |  |  |  |  |  |

**Supplementary Table 4 |**Log-likelihood (logl), degrees of freedom (df) and Akaike Information Criterion (AIC) for the fit to peach/nectarine SSC of multi-trial (F = Fresno, G = College Station, K = Clarksville, S = Seneca) nested univariate (U) or multivariate (M) genotype-by-environment interaction models (G×E mod) of additive (Amod) and dominance (Dmod) genomic effects. Additive genomic environments (AWE) were defined as groupings of trial-by-seasons (where the letter defines the trial and the numbers define the season of assessment) so that within genomic environments it was assumed genomic variance was homogenous, and genomic correlations were 1. Only the interaction between dominance genomic effects and location (DW:L) were tested. Fixed trial-by-season, and random individual tree and tree-by-season residual, effects were also fitted for each trial. The notation *fax* refers to the order (*x*) of a factor analytic structure for the genotype-by-environment variance-covariance matrix.

|  |  |  |  |  |  |  |  |  |  |  |
| --- | --- | --- | --- | --- | --- | --- | --- | --- | --- | --- |
| ID | G×E |  | Amod | AWE | D |  | logl | df | AIC |  |
|  |  |  |  |  |  |  |  |  |  |  |
|  |  |  |  |  |  |  |  |  |  |  |
| MTGWU01 | U |  | AW + AW:AWE1 | F, G, K01, K2, S0, S1, S2 | DW+DW:L |  | -409.7 | 9 | 837.4 |  |
| MTGWU02 | U |  | AW + AW:AWE1 | F, G, K01, K2, S0, S1, S2 | DW |  | -409.7 | 9 | 837.4 |  |
|  |  |  |  |  |  |  |  |  |  |  |
| MTGWM02 | MV |  | AW:*fa1*(AWE1) | F, G, K01, K2, S0, S1, S2 | DW |  | -400.2 | 18 | 836.4 |  |
| MTGWM03 | MV |  | AW:*fa2*(AWE1) | F, G, K01, K2, S0, S1, S2 | DW |  | -397.2 | 23 | 840.4 |  |
| MTGWM05 | MV |  | AW:*fa1*(AWE2) | FK2, G, K01, S0, S1, S2 | DW |  | -403.0 | 19 | 844.0 |  |
| MTGWM06 | MV |  | AW:*fa1*(AWE3) | F, G, K01, K2, S01, S2 | DW |  | -442.0 | 15 | 914.0 |  |
| MTGWM07 | MV |  | AW:*fa1*(AWE4) | F, G, K01S2, K2, S0, S1 | DW |  | -403.0 | 16 | 838.0 |  |
| MTGWM08 | MV |  | AW:*fa1*(AWE5) | FK2, G, K01S2, S0, S1 | DW |  | -405.9 | 17 | 845.8 |  |
| MTGWM09 | MV |  | AW:*fa1*(AWE6) | FG, K01S2, K2, S0, S1 | DW |  | -409.0 | 17 | 852.0 |  |
|  |  |  |  |  |  |  |  |  |  |  |

**Supplementary Table** **5 |**Log-likelihood (logl), degrees of freedom (df) and Akaike Information Criterion (AIC) for the fit to peach/nectarine SSC of multi-trial (F = Fresno, G = College Station, K = Clarksville, S = Seneca) nested univariate (U) or multivariate (M) genotype-by-environment interaction models (G×E mod) of additive and dominance, QTL and background (AQ, AB, DB and DQ, respectively) genomic effects. Additive QTL and background genomic environments (AQE and ABE respectively) were defined as groupings of trial-by-seasons (where the letter defines the trial and the numbers define the season of assessment) so that within genomic environments it was assumed genomic (either QTL or background) variance was homogenous, and genomic correlations were 1. Fixed trial-by-season, and random individual tree and tree-by-season residual, effects were also fitted for each trial. The notation *fax* refers to the order (*x*) of a factor analytic structure for the genotype-by-environment variance-covariance matrix.

|  |  |  |  |  |  |  |  |  |  |  |  |
| --- | --- | --- | --- | --- | --- | --- | --- | --- | --- | --- | --- |
| ID | G×E mod | AQ | AQE | AB | ABE | DQ | DB |  | logl | df | AIC |
|  |  |  |  |  |  |  |  |  |  |  |  |
|  |  |  |  |  |  |  |  |  |  |  |  |
| MTQBU01 | U | AQ + AQ:AQE1 | F, G, K01S2, K2, S0, S1 | AB + AB:ABE1 | F, G, K01S2, K2, S0, S1 | DQ | DB |  | -379.3 | 11 | 781 |
| MTQBU02 | U | AQ + AQ:AQE1 | F, G, K01S2, K2, S0, S1 | AB + AB:ABE1 | F, G, K01S2, K2, S0, S1 |  | DB |  | -379.3 | 11 | 781 |
| MTQBU03 | U | AQ + AQ:AQE1 | F, G, K01S2, K2, S0, S1 | AB + AB:ABE1 | F, G, K01S2, K2, S0, S1 |  |  |  | -382.7 | 10 | 785 |
| MTQBU04 | MV | AQ |  | AB + AB:ABE1 | F, G, K01S2, K2, S0, S1 |  | DB |  | -380.9 | 10 | 782 |
| MTQBU05 | MV | AQ + AQ:AQE1 | F, G, K01S2, K2, S0, S1 | AB |  |  | DB |  | -421.8 | 11 | 866 |
|  |  |  |  |  |  |  |  |  |  |  |  |
| MTQBMB1 | MV | AQ |  | fa1(ABE1):AB | F, G, K01S2, K2, S0, S1 |  | DB |  | -370.7 | 18 | 777 |
| MTQBMB2 | MV | AQ |  | fa1(ABE2):AB | FK2, G, K01S2, S0, S1 |  | DB |  | -374.6 | 19 | 787 |
| MTQBMB3 | MV | AQ |  | fa1(ABE3):AB | FG, K01S2, K2, S0, S1 |  | DB |  | -375.2 | 18 | 786 |
|  |  |  |  |  |  |  |  |  |  |  |  |

**Supplementary Table** **6 |** Estimated additive (A) and total genomic (G = additive + dominance ) correlations for genome wide (W), QTL (Q) or background genomic effects across genomic environments (GEnv, defined as a group of trials-by-seasons across which it is assumed genomic – genome-wide , QTL or background genomic – variance were homogeneous and correlations were 1 where letters define trial and numbers define year of assessment, see Table 1a for detail) for most parsimonious multivariate multi-trial models of peach/nectarine SSC **Supplementary Tables** **4** and **5**).

|  |  |  |  |  |  |  |  |  |  |  |  |
| --- | --- | --- | --- | --- | --- | --- | --- | --- | --- | --- | --- |
| Effect |  |  |  |  |  |  |  |  |  |  |  |
|  | GEnv |  | G |  | K01S2 |  | K2 |  | S0 |  | S1 |
|  |  |  |  |  |  |  |  |  |  |  |  |
|  |  |  |  |  |  |  |  |  |  |  |  |
| GQB | F |  | 0.81 |  | 0.66 |  | 0.80 |  | 0.16 |  | 0.22 |
| GW | F |  | 0.80 |  | 0.58 |  | 0.81 |  | 0.38 |  | 0.37 |
|  |  |  |  |  |  |  |  |  |  |  |  |
| AQB | F |  | 0.80 |  | 0.61 |  | 0.78 |  | 0.06 |  | 0.11 |
| AB | F |  | 0.74 |  | 0.46 |  | 0.69 |  | -0.33 |  | -0.27 |
| AW | F |  | 0.78 |  | 0.50 |  | 0.78 |  | 0.25 |  | 0.24 |
|  |  |  |  |  |  |  |  |  |  |  |  |
| GQB | G |  |  |  | 0.75 |  | 0.95 |  | 0.06 |  | 0.13 |
| GW | G |  |  |  | 0.68 |  | 1.00 |  | 0.42 |  | 0.40 |
|  |  |  |  |  |  |  |  |  |  |  |  |
| AQB | G |  |  |  | 0.72 |  | 0.94 |  | -0.05 |  | 0.02 |
| AB | G |  |  |  | 0.62 |  | 0.94 |  | -0.45 |  | -0.37 |
| AW | G |  |  |  | 0.64 |  | 1.00 |  | 0.32 |  | 0.31 |
|  |  |  |  |  |  |  |  |  |  |  |  |
| GQB | K01S2 |  |  |  |  |  | 0.77 |  | 0.28 |  | 0.33 |
| GW | K01S2 |  |  |  |  |  | 0.70 |  | 0.37 |  | 0.36 |
|  |  |  |  |  |  |  |  |  |  |  |  |
| AQB | K01S2 |  |  |  |  |  | 0.73 |  | 0.17 |  | 0.22 |
| AB | K01S2 |  |  |  |  |  | 0.58 |  | -0.28 |  | -0.23 |
| AW | K01S2 |  |  |  |  |  | 0.64 |  | 0.20 |  | 0.20 |
|  |  |  |  |  |  |  |  |  |  |  |  |
| GQB | K2 |  |  |  |  |  |  |  | 0.20 |  | 0.26 |
| GW | K2 |  |  |  |  |  |  |  | 0.45 |  | 0.44 |
|  |  |  |  |  |  |  |  |  |  |  |  |
| AQB | K2 |  |  |  |  |  |  |  | 0.07 |  | 0.14 |
| AB | K2 |  |  |  |  |  |  |  | -0.43 |  | -0.35 |
| AW | K2 |  |  |  |  |  |  |  | 0.32 |  | 0.31 |
|  |  |  |  |  |  |  |  |  |  |  |  |
| GQB | S0 |  |  |  |  |  |  |  |  |  | 0.54 |
| GW | S0 |  |  |  |  |  |  |  |  |  | 0.30 |
|  |  |  |  |  |  |  |  |  |  |  |  |
| AQB | S0 |  |  |  |  |  |  |  |  |  | 0.47 |
| AB | S0 |  |  |  |  |  |  |  |  |  | 0.17 |
| AW | S0 |  |  |  |  |  |  |  |  |  | 0.10 |
|  |  |  |  |  |  |  |  |  |  |  |  |

## Figures


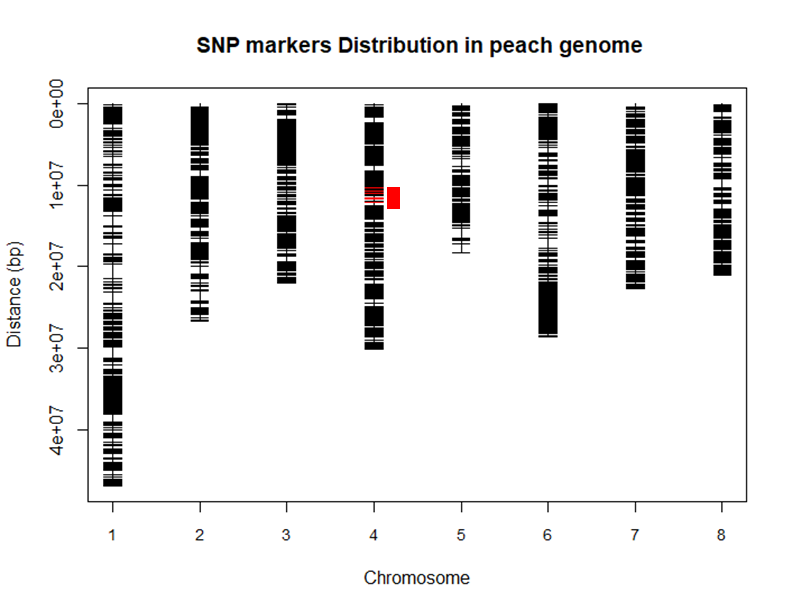


**Supplementary Figure** **1 |** Distribution of physical location on the peach reference genome of anonymous SNP markers (black) and region of eight SNP markers containing previously reported (Eduardo et al., 2011) QTL for SSC (red) used in this study to model genomic variation in peach/nectarine SSC.


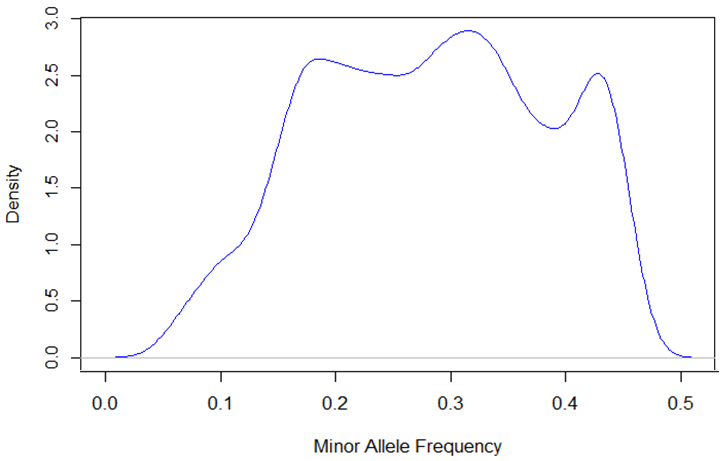


**Supplementary Figure** **2 |** Distribution of minor allele frequency across 577 peach/nectarine individuals included in this study to model genomic variation on SSC,

| TAMU  Cross population  Within population | Cross population  Within population |
| --- | --- |
| UARK  Cross population  Within population | UARK  Within population  Cross population |
| CLEM  Cross population  Within population | CLEM  Cross population  Within population |

**Supplementary Figure** **3 |** Plots of (a) allele frequencies of loci for three individual peach/nectarine populations (TAMU = Texas A & M University, UARK = University of Arkansas, CLEM = Clemson University) by allele frequency across populations, and (b) pair-wise additive genomic relationships among individuals estimated using only within population allele frequencies elements by relationships among the same individuals estimated using across population frequencies.


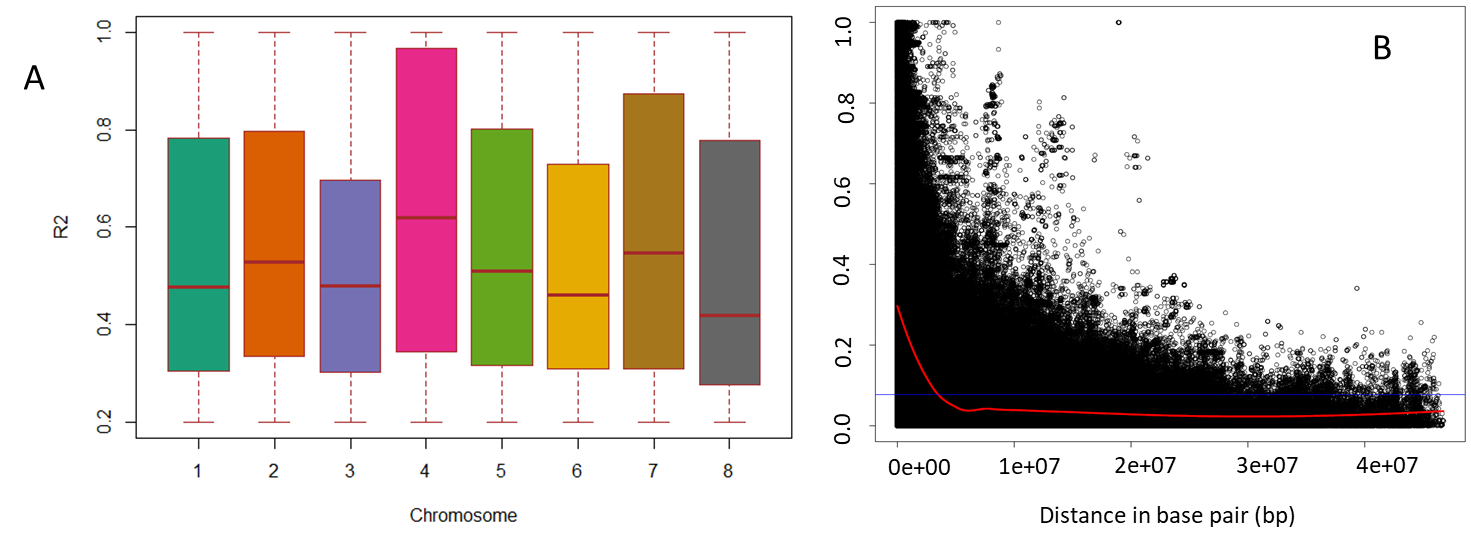
**Supplementary Figure 4 |** Distribution of pairwise linkage disequilibrium (R^2^) by chromosome across peach/nectarine populations in this study.

**Supplementary Figure** **5a |** Cluster dendrogram of total genomic correlation matrix across genomic environments (F = Fresno 2011 and 2012, G = College Station 2012 and 2013, K01S2 = Clarksville 2010 and 2011 and Seneca 2012, S0 = Seneca 2010, S1 = Seneca 2011) estimated from the most parsimonious genome-wide model (MTGWM07, **Supplementary Table** **4**) for SSC assessed across four peach/nectarine breeding trials. Genomic environments are defined as groupings of trial-by-seasons such that genomic variance is homogeneous, and genomic correlations are 1, within environments.

**Supplementary Figure** **5b |** Cluster dendrogram of additive genomic correlation matrix across genomic environments (F = Fresno 2011 and 2012, G = College Station 2012 and 2013, K01S2 = Clarksville 2010 and 2011 and Seneca 2012, S0 = Seneca 2010, S1 = Seneca 2011) estimated from the most parsimonious QTL+ background genomic model (MTQBMB1, **Supplementary Table** **5**) for SSC assessed across four peach/nectarine breeding trials. Genomic environments are defined as groupings of trial-by-seasons such that genomic variance is homogeneous, and genomic correlations are 1, within environments.

**Supplementary Figure** **5c |** Cluster dendrogram of additive background genomic correlation matrix across genomic environments (F = Fresno 2011 and 2012, G = College Station 2012 and 2013, K01S2 = Clarksville 2010 and 2011 and Seneca 2012, S0 = Seneca 2010, S1 = Seneca 2011) estimated from the most parsimonious QTL+ background genomic model (MTQBMB1, **Supplementary Table** **5**) for SSC assessed across four peach/nectarine breeding trials. Genomic environments are defined as groupings of trial-by-seasons such that genomic variance is homogeneous, and genomic correlations are 1, within environments.

**Supplementary Figure** **5b |** Cluster dendrogram of additive genome-wide correlation matrix across genomic environments (F = Fresno 2011 and 2012, G = College Station 2012 and 2013, K01S2 = Clarksville 2010 and 2011 and Seneca 2012, S0 = Seneca 2010, S1 = Seneca 2011) estimated from the most parsimonious genome-wide model (MTGWM07, **Supplementary Table** **4**) for SSC assessed across four peach/nectarine breeding trials. Genomic environments are defined as groupings of trial-by-seasons such that genomic variance is homogeneous, and genomic correlations are 1, within environments.
